# Revealing Non-ketotic Hyperglycemia as a Trigger for Hemichorea-Hemiballismus in Uncontrolled Diabetic Asthmatics: A Case Report

**DOI:** 10.7759/cureus.55678

**Published:** 2024-03-06

**Authors:** Pranav Chaudhari, Rucha Sawant, Vineet Karwa, Sarang S Raut, Sourya Acharya, Sunil Kumar

**Affiliations:** 1 Medicine, Jawaharlal Nehru Medical College, Wardha, IND

**Keywords:** steroid, hemiballismus, chorea disorders, diabetes mellitus, asthma, hyperglycemia

## Abstract

Uncontrolled diabetes can trigger a movement disorder called hemichorea-hemiballismus, characterized by non-ketotic hyperglycemia-related chorea/ballism and usually reversible basal ganglia abnormalities on CT and/or MRI. The condition is diagnosed clinically and is mostly based on radiological imaging. Here, we report a case of a 68-year-old female presenting with right-sided and facial involuntary movements owing to uncontrolled hyperglycemia who was treated with antidiabetic and anticholinergic medications. The patient responded well to the treatment and showed a favorable outcome with no complications.

## Introduction

Quick, rapid, and erratic motions typify the unusual involuntary movement condition known as chorea. Chorea can stem from a variety of conditions, including neurodegenerative disorders, cerebrovascular disorders, immunological causes, neoplasia, infectious disease, and metabolic dysfunction [1]. Hemiballismus is the more common form of ballismus, affecting only one side of the body with violent, flinging movements of the extremities. Ballismus, in rare cases, can involve both sides of the body; it is a dramatic movement disorder and shares similar underlying causes with chorea. People with poorly controlled type 2 diabetes may experience “chorea-ballismus,” a rarely encountered movement disorder known as non-ketotic hyperglycemia (NKH) chorea [2].

## Case presentation

A female in her late 60s presented with a four-day history of regular, rhythmic, involuntary movements of her right limbs, mouth, and tongue (Video 1). This was present only when the patient was awake and absent during sleep. Upon detailed history, it was elicited that she had diabetes mellitus for nine years and was on treatment with metformin 1 g twice a day and glimepiride 2 mg twice a day. She was generally compliant with medication but had not visited a physician for the same for the last three years. Additionally, she had a history of mild intermittent asthma and experienced a moderate exacerbation 14 days ago. She was treated at a primary center with levosalbutamol nebulization four hours a day, and oral prednisolone 40 mg was initiated the next day due to persistent symptoms. The symptoms improved the following day, and despite the recommendation for further investigations for diabetes control, as her random blood sugar (RBS) was 268 mg/dl, she was not willing and was discharged upon request.

**Video 1:** This shows regular, rhythmic, involuntary movements of the right upper limb, mouth, and tongue at the time of presentation

Over the last seven days, the patient developed a tingling sensation over the right hand and legs, which eventually progressed to the entire upper and lower limbs, which the patient ignored. In the last four days, she developed involuntary movement of her right limbs, which eventually progressed to her mouth and tongue over three days. The patient could not eat for one day when she went to a local practitioner. An MRI brain was performed, which was reported to be normal, and she was referred to a higher center for further management.

**Figure 1:**
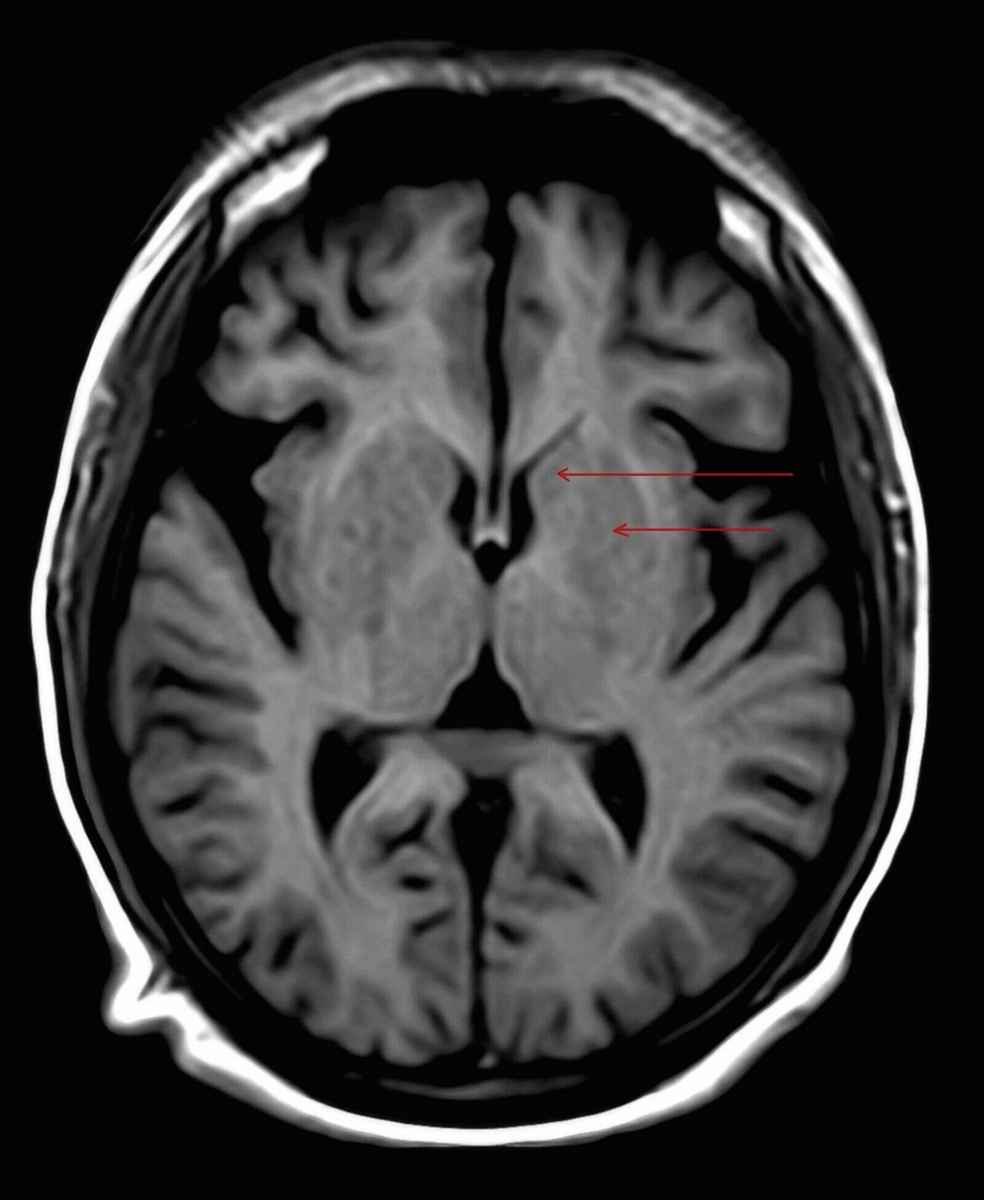
This shows an MRI T1-weighted image with no obvious abnormality in the basal ganglia (red arrows showing basal ganglia)

**Figure 2:**
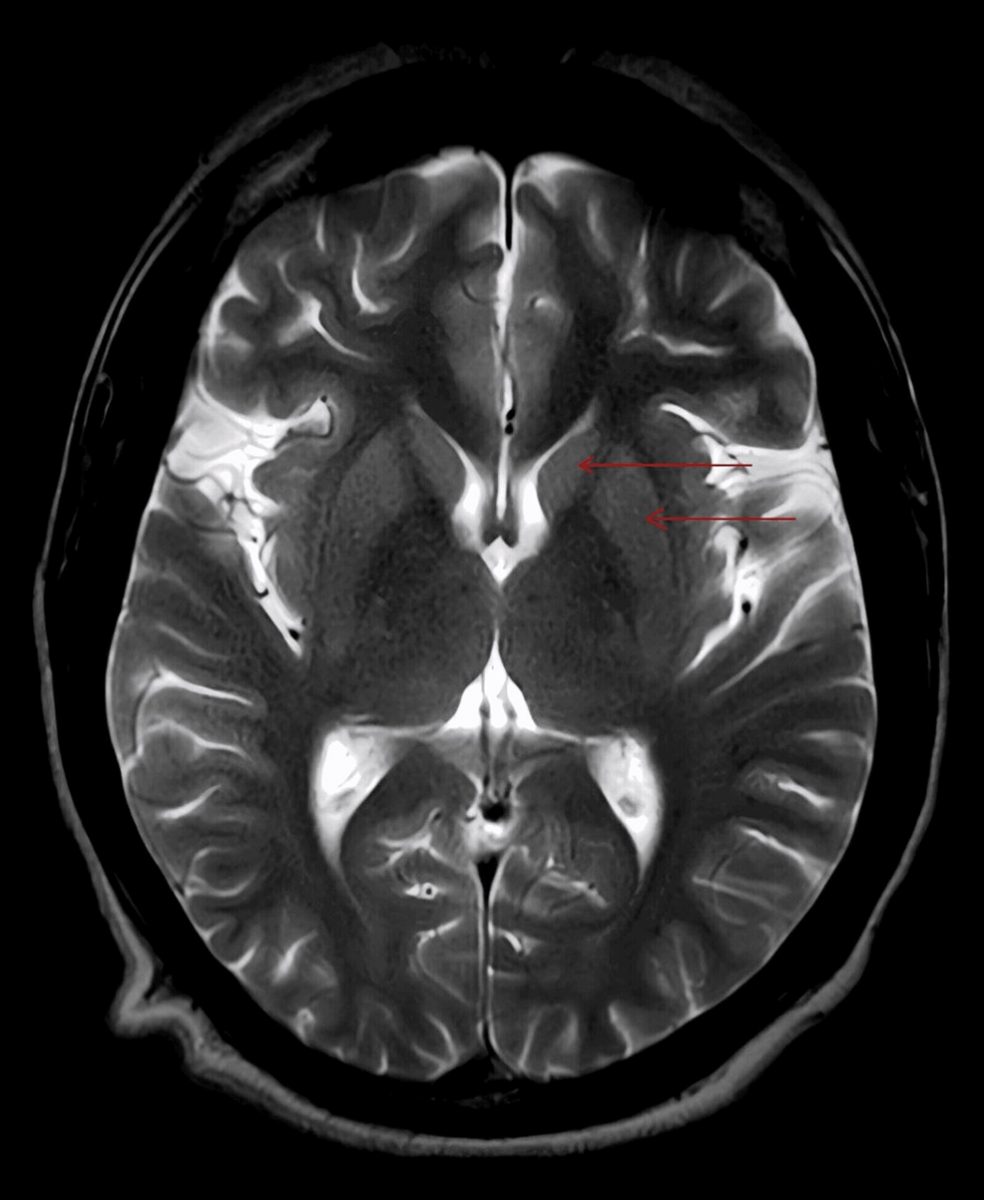
This shows an MRI T2-weighted image with no obvious abnormality in the basal ganglia (red arrows showing basal ganglia)

**Figure 3:**
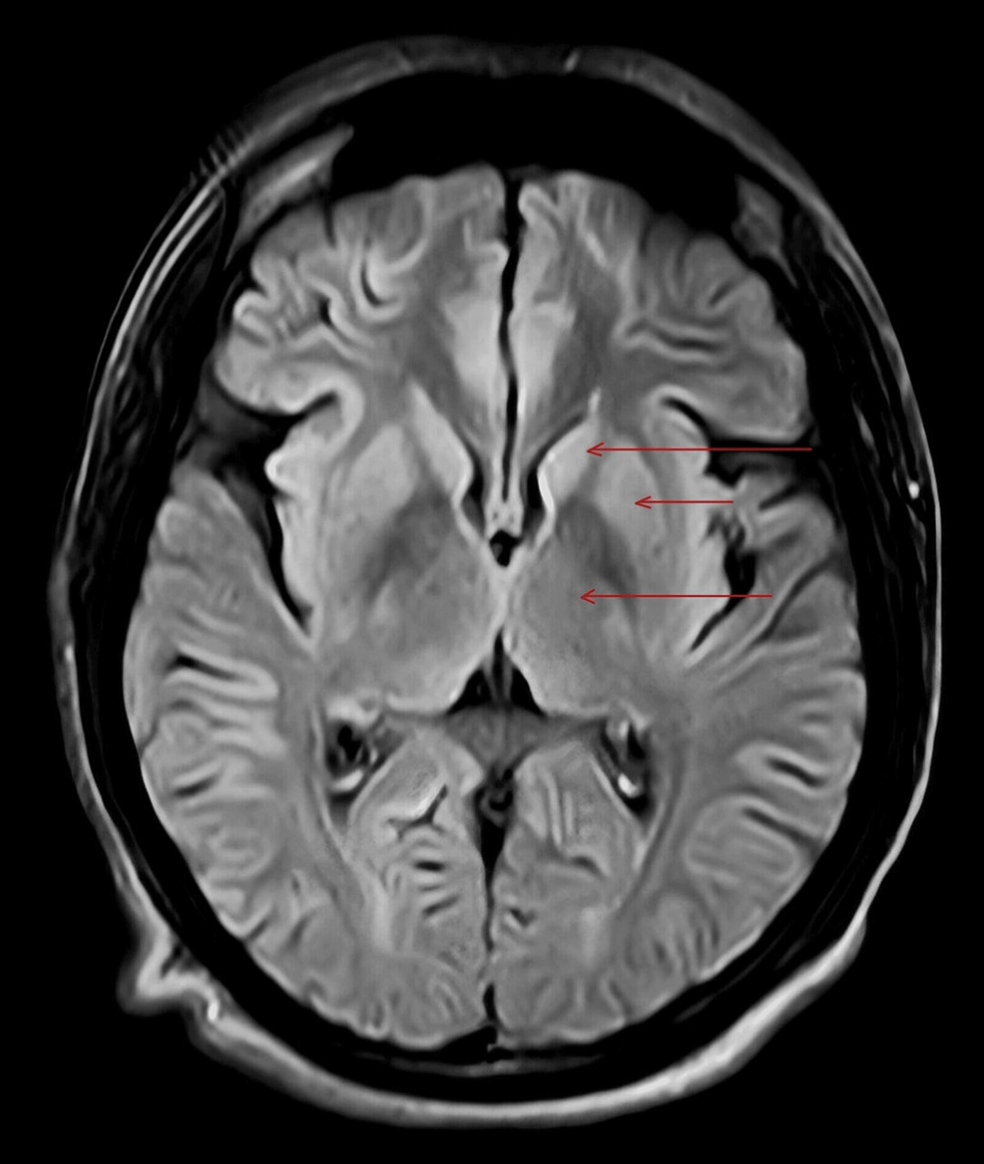
This shows an MRI FLAIR image with no obvious abnormality in the basal ganglia (red arrows showing basal ganglia)

Upon presentation, her vitals were normal. During examination, she exhibited mild tenderness over the right upper quadrant and abnormal movements in her right limbs and mouth. Her RBS, tested at that time, was 318 mg/dl; urine sugar was 3+, urine ketones were negative, and glycosylated hemoglobin was >14%. Other laboratory reports, including liver and kidney function tests, thyroid profile, antinuclear antibody, antineutrophilic cytoplasmic antibodies (Cytoplasmic (C) and Perinuclear (P) antineutrophil cytoplasmic antibodies), serum ceruloplasmin, HIV test, HbsAg test, vitamin B12, and vitamin D, were unremarkable (Table 1). The MRI brain, reviewed by the radiologist and neurologist, did not reveal any abnormalities in the basal ganglia (Figure 1, Figure 2, and Figure 3). There was no significant history of any drug intake other than antidiabetics, antiasthma drugs, and oral steroids. The patient was diagnosed with hemichorea-hemiballismus (HC-HB) associated with NKH after ruling out other metabolic causes of chorea such as hypomagnesemia, hypoglycemia, hyponatremia, hypernatremia, hypocalcemia, and vitamin B12 deficiency. The patient was started on an insulin sliding scale and received symptomatic treatment for chorea with tetrabenazine 25 mg twice a day, clonazepam 0.5 mg at night, and haloperidol 0.5 mg at night. Within two days of hospitalization, her symptoms improved dramatically, and her blood sugar levels normalized with negative urine sugar. After seven days, her involuntary movements completely disappeared. Upon discharge, she was prescribed formoterol (4.5 mcg) and budesonide (160 mcg) metered-dose inhalers for use as needed, which she used only once post-discharge. Over three months, tetrabenazine, clonazepam, and haloperidol were tapered off, and blood sugar was controlled using a combination of insulin glargine and oral antidiabetics (metformin, glimepiride, and teneligliptin). After six months of follow-up, she showed no abnormality, and her blood sugar levels were controlled with a glycosylated hemoglobin of 6.2%. The patient declined follow-up radiological imaging.

**Table 1:** Laboratory investigations ANA, antinuclear antibody; ANCA, antineutrophil cytoplasmic antibodies; HbA1C, hemoglobin A1C; INR, international normalized ratio; MPO, myeloperoxidase; PR3, proteinase 3; RBS, random blood sugar; TSH, thyroid-stimulating hormone

Parameters	Values	Normal range
Hemoglobin(gm/dl)	12.1	Nov-15
Total white blood cell count (per cu. mm)	6,600	4,000-10,000
Total red cell count (million per cu .mm)	4.4	4.2-5.4
Platelets (per cu. mm)	273,000	140,000-440,000
Hematocrit (%)	42	36-46
Urea (gm/dl)	44	Oct-45
Creatinine (mg/dl)	1.2	0.2-1.2
Sodium (meq/l)	138	135-148
Potassium (meq/l)	4.4	3.5-5.3
Magnesium (mg/dl)	2.1	1.6-2.3
Calcium (mg/dl)	8.9	8.4-10.2
Phosphorus (mg/dl)	4.8	2.5-4.5
Uric acid (mg/dl)	5.2	2.5-6.5
RBS (mg/dl)	318	<140
Serum glutamyl-oxaloacetate transaminase (U/l)	36	11.8-64.8
Serum glutamyl pyruvate transaminase (U/l)	18	8.5-49.5
Albumin (gm/dl)	3.9	3.2-5.1
Total bilirubin (gm/dl)	0.5	0-0.8
INR	1.05	1.3-1.5
HbA1c (%)	14	<6
Vitamin D (ng/ml)	28	>30
Vitamin B12 (pg/ml)	865	239-913
Serum ceruloplasmin (mg/dl)	24	20-40
ANA	0.62	0-0.9: negative; 0.9-1.1: borderline; positive: >1.1
PR3-ANCA (C-ANCA) (u/ml)	1.7	Negative <5
MPO-ANCA (P-ANCA) (u/ml)	3.4	Negative <5
TSH (µIU/ml)	4.34	0.465-4.68
fT3 (pg/ml)	3.9	3.77-5.27
fT4 (ng/ml)	0.9	0.78-2.19

## Discussion

NKH HC-HB is a rare entity in the context of diabetes or acute hyperglycemia [3]. A standard presentation of patients with NKH HC-HB involves a triad of symptoms: (1) NKH, (2) HC, and (3) MRI T1 showing a high signal or CT scan demonstrating high density in the basal ganglia [4]. NKH HC constitutes an unusual clinical disease initially documented by Bedwell in 1960 [5]. Examining 53 cases from 1985 to 2001, researchers found chorea and/or ballismus as movement disorders associated with hyperglycemia. The predominantly Asian group (91%) had an average age of 71.1 years, with a male-to-female ratio of 1:1.76. Variations in movement disorder susceptibility among different racial and gender groups might be linked to both genetic factors and estrogen levels. When estrogen levels decline after menopause, the dopamine receptors in the nigrostriatal pathway become more sensitive, potentially leading to an overactive dopamine system within the striatum.

Usually, involuntary movement occurs before the diagnosis of diabetes mellitus, contrary to our case. Most patients with NKH HC-HB exhibit characteristic hyperdense lesions in the basal ganglia on cranial CT scans, while a few others show no abnormalities. Frequently, MRI brain shows increased signal intensity, specifically in the putamen and caudate nucleus, particularly on the T1-weighted sequence [6]; our case showed no obvious abnormality on MRI brain, thus making this a unique, rare case and providing further understanding of this disease entity.

In most NKH-related chorea cases, patients experience a sudden or gradual onset of involuntary limb movements, typically affecting one side of the body. These movements can also involve facial muscles, the jaw, and the tongue. This occurs alongside significantly high blood sugar levels and absent ketones in urine. As the patient’s condition improves, the associated brain imaging abnormalities may improve or even resolve entirely. It is worth noting that relatively few patients present with negative results on either CT or MRI scans [1,7,8]. With an early diagnosis, most patients have a good prognosis in NKH HC-HB. Managing blood sugar levels is essential for treatment and may even reduce uncontrollable movements for some patients. In more severe cases, medications like clonazepam, sometimes in combination with drugs antagonizing dopamine receptors like haloperidol or risperidone, may be used to manage chorea.

Prednisolone accelerates the breakdown of proteins, decreases the metabolism of glucose, and initiates peripheral lipolysis. This process provides amino acids and glycerol to support gluconeogenesis, leading to elevated blood glucose levels [9]. Due to these combined effects of glucocorticoids (GCs) on both insulin sensitivity and islet cell function and their specific pharmacokinetic profile, it has become clear that synthetic GCs particularly increase postprandial glucose levels [10] without affecting fasting glucose levels. Underestimating steroid-induced diabetes is a concern, as many healthcare centers primarily monitor fasting glucose during treatment. Surprisingly, despite the established link between GCs and diabetes, research on optimal treatment and, ideally, prevention of GC-induced diabetes is scarce.

The precise mechanism of NKH HC-HB remains a mystery. One possible explanation involves hyperglycemia causing a shift in brain cell metabolism from aerobic (oxygen dependent) to anaerobic (oxygen independent) processes due to reduced blood flow and compromised glucose utilization. This switch could make gamma-aminobutyric acid (GABA) the primary energy source, relying on acetoacetate, readily available in ketosis patients, for its synthesis. In NKH, there is evidence suggesting reduced levels of GABA, which is thought to be related to a lack of acetoacetate that contributes to impaired activity in the basal ganglia [2,11,12]. Only a few instances of negative imaging results have been reported in the literature [13,14]. Although most patients recover well after blood sugar control is achieved, up to 20% may continue to experience symptoms [13]. In these situations, symptoms-focused management is necessary, but no controlled studies or treatment algorithms are currently available.

## Conclusions

This case underscores the rare but crucial role of NKH in triggering movement disorders like HC-HB. After diligently ruling out more common causes, clinicians should consider NKH by reviewing the patient’s history and performing basic blood tests. When using steroids in a diabetic patient, the diabetogenic effect of GCs should be kept in mind, the fasting and postprandial glucose profiles of the patient should be monitored, and more tailored therapy should be initiated. An analysis of existing cases, including ours, suggests two potential subtypes of NKH-associated HC-HB. The most common type affects individuals with diabetes and is characterized by elevated blood sugar, absent ketones, and unilateral or bilateral chorea. These patients often exhibit characteristic brain imaging changes on MRI or CT scans. Less common is a subtype presenting similarly to diabetes, hyperglycemia, and negative ketones but without detectable abnormalities on brain imaging despite the presence of chorea. Since NKH HC-HB is a treatable condition, its recognition is important.
